# Beyond Shape: How You Learn about Objects Affects How They Are Represented in Visual Cortex

**DOI:** 10.1371/journal.pone.0008405

**Published:** 2009-12-22

**Authors:** Alan C.-N. Wong, Thomas J. Palmeri, Baxter P. Rogers, John C. Gore, Isabel Gauthier

**Affiliations:** 1 Department of Psychology, The Chinese University of Hong Kong, Shatin, Hong Kong; 2 Department of Psychology, Vanderbilt University, Nashville, Tennessee, United States of America; 3 Institute of Imaging Science, Vanderbilt University, Nashville, Tennessee, United States of America; Victoria University of Wellington, New Zealand

## Abstract

**Background:**

Experience can alter how objects are represented in the visual cortex. But experience can take different forms. It is unknown whether the kind of visual experience systematically alters the nature of visual cortical object representations.

**Methodology/Principal Findings:**

We take advantage of different training regimens found to produce qualitatively different types of perceptual expertise behaviorally in order to contrast the neural changes that follow different kinds of visual experience with the same objects. Two groups of participants went through training regimens that required either subordinate-level individuation or basic-level categorization of a set of novel, artificial objects, called “Ziggerins”. fMRI activity of a region in the right fusiform gyrus increased after individuation training and was correlated with the magnitude of configural processing of the Ziggerins observed behaviorally. In contrast, categorization training caused distributed changes, with increased activity in the medial portion of the ventral occipito-temporal cortex relative to more lateral areas.

**Conclusions/Significance:**

Our results demonstrate that the kind of experience with a category of objects can systematically influence how those objects are represented in visual cortex. The demands of prior learning experience therefore appear to be one factor determining the organization of activity patterns in visual cortex.

## Introduction

How does experience with objects such as dogs, roses or cars change their representation in visual cortex? Practice with objects is accompanied by increased activity [1]–[5] and increased sensitivity in visual areas to subtle differences in shape [6], [7]. But our experience with objects can take many different forms: We can be passively exposed to objects, learn to categorize them into different groups, learn unique names or other meaningful information about them, or learn to discriminate between them without ever naming them. Do visual representations depend on the kind of experience we have had with objects, or does any kind of experience lead to similar cortical representations of object shapes, perhaps only quantitatively modulated by frequency of exposure and amount of visual attention? To date, studies have mainly demonstrated that experience matters. Relatively few studies have shown systematically that the kind of experience we have with objects matters for determining the nature of visual cortical representations.

Functional brain imaging studies manipulating object category have revealed neural activity patterns in the ventral occipito-temporal cortex (VOT) specific for familiar visual categories. Some categories (e.g., faces, body parts, places) display strong selectivity that is clustered in one or a few local areas [8]–[13]; but for the majority of categories, selectivity is weaker and emerges from pooling the responses distributed over larger expanses of visual cortex [14]. Some categories, such as letters, show both clustered and distributed activity [15], [16].

Such category-selective activity patterns imply a number of candidate factors affecting object representations in visual cortex. When perception of animals and tools, faces and houses, or letters and digits are contrasted, differences can be attributed to a host of confounded factors, like differences in shape, name, meaning, frequency, and history of processing. One possibility is that object processing areas in visual cortex contain a large-scale topographical representation based on object shape [17] that is not qualitatively modified by experience [18]–[21] but only quantitatively fine-tuned. However, perceptual expertise studies with categories as diverse as letters [10], musical notation [22], cars and birds [23] suggest that experience plays an important role in determining the different neural representations observed in various domains.

We investigated whether the kind of experience with an object category significantly determines how objects are represented in visual cortex, controlling for object shape. Specifically, experience with discrimination at different levels of abstraction was examined. A number of studies have looked at the neural correlates of object classification at different levels with mixed results. For example, Gauthier *et al.* [24] used a label-picture matching task and found within a wide range of regions in VOT higher activity for subordinate- than basic-level judgment (e.g., matching an image to the label “robin” vs. “bird”). Op de Beeck et al. [25] asked participants to match objects in a basic-level category or at the exemplar level and found no activity difference in VOT. Tyler *et al.* [26] asked participants to silently name a number of common objects and found that the anterior regions of the inferior temporal cortex were more active during basic-level naming (e.g., “monkey”) and the posterior regions were more active during domain-level naming (“living thing”). It can be difficult to specifically pinpoint the cause of such discrepancies in studies using a range of real-world object categories. Since common objects are most often categorized at the basic level, a subordinate-level categorization might impose a more unfamiliar task demand leading to more brain regions being activated [as in 24]. In addition, common objects have associated with them numerous semantic attributes, which may also affect object representations in the VOT [8]. Depending on the exact object categories used in an experiment, different results may be obtained due to different semantic attributes becoming activated. Because of all of these complications with real-world objects, in order to fully understand how the kind of experience with objects affects their representations in visual cortex, we argue it is best to use novel objects that participants have never seen before.

Surprisingly, while different object shapes and different tasks are very often contrasted in neuroimaging studies of object recognition, including those described above, there has been far less effort in contrasting different kinds of learning experience with objects. While some work has compared subordinate-level categorization training with a control training equating for exposure [7], [27]–[29], the comparison training has typically been very easy and involved considerably lower degrees of learning. Thus, any differences observed in neural activity could be caused by either different levels of attention or different degrees of expertise.

Here, we tested how different kinds of learning experience influence visual cortical representations by contrasting two difficult training protocols that have been shown to result in qualitatively different kinds of object learning [30]. The same set of objects was learned in two different ways by two groups of participants. By doing so, we manipulate learning history for those objects, holding shape constant. Before and after learning, participants were tested in the same way. Brain activity for both groups was measured after learning under the same testing conditions.

**Figure 1:**
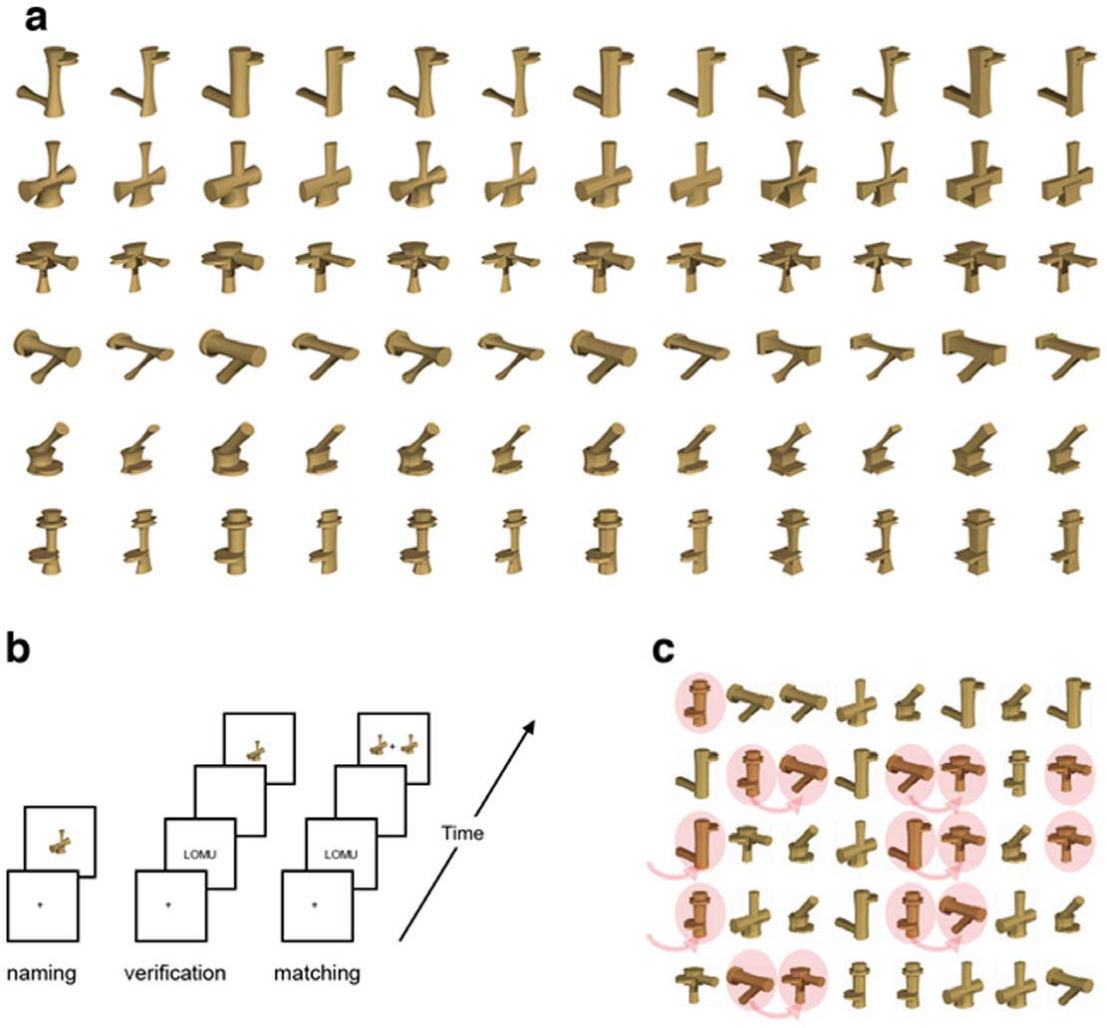
The artificial objects (Ziggerins) and training tasks. (a). The entire Ziggerin set of seventy-two novel objects. The six classes were shown in separate rows. (b). Individuation training consisted of three tasks: naming, verification, and matching. (c). Categorization training consisted of the naming and verification tasks, as well as a matrix scanning task with an example trial shown.

Using the same set of novel artificial objects (called Ziggerins), we modeled our two training regimens after the sort of experience thought to underlie face individuation and letter categorization, respectively (see Fig. 1). Individuation training focused on rapid identification of objects that share a common part structure [31]. Categorization training required participants to rapidly distinguish objects with different structures in arrays where objects shared the same style, much like the common size, orientation, and font of different letters on a page [32]. These two training regimens resulted in qualitatively different behavioral changes [30]: Individuation training increased holistic processing selectively for Ziggerins in the trained configuration and speeded up individuation of the Ziggerins. In contrast, after training, the categorization–training group was faster than the individuation-training group in basic-level recognition of Ziggerins either appearing in isolation or three at a time.

**Figure 2:**
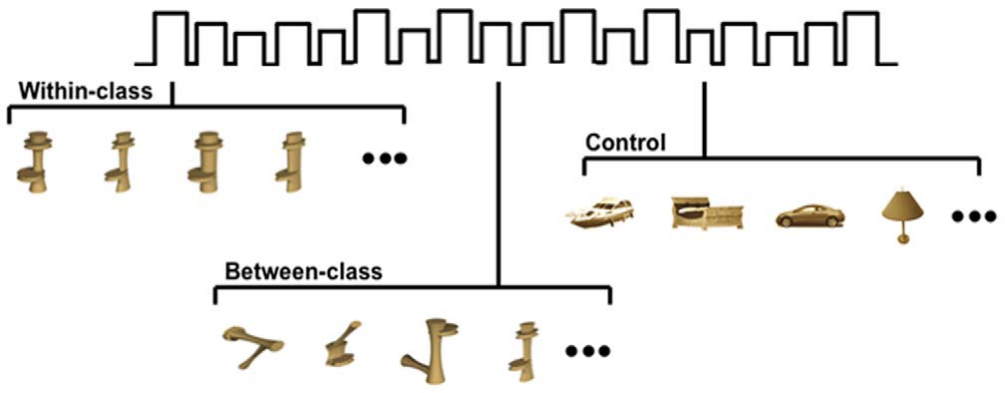
An example scan of the fMRI pre- and post-training scans. In the within-class discrimination condition, each block contained only Ziggerins within the same class, thus requiring within-class/subordinate-level discrimination. In the between-class discrimination condition, each block contained Ziggerins from different classes, thus requiring between-class/basic-level discrimination. The control blocks contained familiar objects from different categories.

Using functional magnetic resonance imaging (fMRI), we now ask whether the two training regimens also produce different patterns of changes in visual cortex (Fig. 2). The testing tasks within the scanner were the same for both groups and involved both subordinate-level identification and basic-level categorization, to allow each training regimen to reveal their influences.

## Results

Two groups of participants learned to either individuate or categorize a set of novel objects (Ziggerins) in ten 1-hour sessions. Functional magnetic resonance imaging (fMRI) responses to the Ziggerins were measured before and after training. During the fMRI sessions participants performed either within- or between-category discriminations (see Methods). Behavioral performance within the scanner was generally better for all participants after training, with no significant difference between the two training groups (see Supporting Information S1).

For fMRI, we computed the training effects by subtracting the activity for Ziggerins (relative to object control) before training from that after training. A positive (or negative) training effect would thus index an increase (or decrease) in brain activity after training. As the individuation and categorization training were inspired by our experience in face and letter recognition respectively, we asked if the two training regimens would selectively increase the activity in face- and letter selective regions. We also considered changes more broadly in the ventral occipito-temporal cortex.

### Face- and Letter-Selective Regions

Using data from the localizer scans, we identified at the group level a region in the right fusiform gyrus with higher activity for faces than letters and objects (Talairach coordinates: 40, −44, −18; size = 462 mm^3^), and a region in the left inferior temporal gyrus with higher activity for letters than faces and objects (coordinates: −50, −46, −6; size = 844 mm^3^). As control regions, two areas in bilateral parahippocampal cortices were identified (coordinates: −30, −36 −15 (left) and 27, −45, −8 (right); size = 1000 mm^3^ for both) that responded more to objects than faces [33].

**Figure 3:**
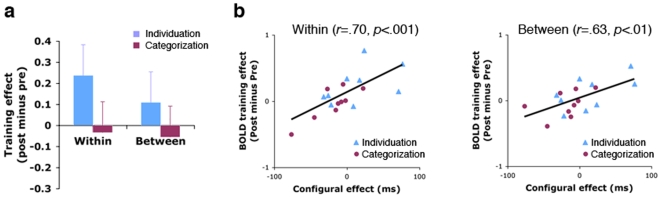
Training effects in the group-defined face-selective, right fusiform region (Talairach coordinates: 40, −44, −18). (a). Activity changes (post minus pre) for Ziggerins during within- and between-class discrimination after individuation vs. categorization training. Error bars represent the 95% confidence intervals for group differences. (b). Correlations between training-induced activity changes in this right fusiform region for the Ziggerins and the configural processing effect found for the Ziggerins measured behaviorally.

The right fusiform region showed increased activity for the Ziggerins in the individuation group after training (Fig. 3a). A repeated-measures ANOVA on the training effect with Group (individuation vs. categorization training) and Task (within- vs. between-class discrimination) as factors revealed a larger increase in activity for the individuation-training than the categorization-training group [*F*_1,16_ = 4.93, *P* = .041]. The training effect in this region was significant in the individuation-training group for within-class discrimination [*t*_8_ = 2.50, *P* = .036] but did not reach significance for between-class discrimination [*P* = .223]. No significant training effect was found in the categorization-training group for either task [*t*s<1]. The letter- and object-selective regions showed no significant training effect either (*t*s<1).

We also observed a correlation between the training effect in this right fusiform region and the extent to which Ziggerins were processed configurally after training (Fig. 3b). We previously reported data from a Ziggerin composite task conducted outside the scanner in which participants matched half of a Ziggerin image while trying to ignore the other half [30]. Individuation experts were less able to ignore an irrelevant part, especially when the Ziggerins appeared in their trained configuration, i.e., the two halves were aligned with one another. Here, we found that this configural effect (see [30] for details) was correlated with the magnitude of the training effect in the face-selective region across all participants [Pearson product-moment correlations: within: *r* = .70, *P*<.001; between: *r* = .63, *P* = .005]. While the large, negative configural effects for two participants in the categorization-training group (the two leftmost data points in Fig. 3b) were hard to interpret, the correlations remained significant after the two data points were discarded [within: *r* = .53, *P* = .03; between: *r* = .59, *P* = .02].Participants in the individuation-training group tended to have larger configural effects and larger training effects than participants in the categorization-training group. The correlations remained high with separate analyses for the categorization-training [within: *r* = .82, *P* = .006; between: *r* = .49, *P* = .185] and individuation-training groups [within: *r* = .50, *P* = .167; between: *r* = .60, *P* = .084]. There were no significant correlations of this task with activity in letter- and object-selective regions.

Caution should be taken in interpreting the significant training effects in the right fusiform region defined at the group level as evidence for the development of face-like selectivity for Ziggerins. For the face-selective fusiform region localized in individual participants (i.e., the right fusiform face area, or rFFA), training effect did not reach significance (*t*<1) in the individuation-training group and had a weak correlation with configural processing of Ziggerins [within: *r* = .33, *P* = .177; between: *r* = .43, *P* = .073]. Although the face-selective region defined at the group vs. individual levels overlapped considerably (coordinates: 40, −44, −18 vs. 38.9, −43.4, −14.6; see Supporting Information S1), the discrepancy of results reveals that the training effect associated with individuation training and configural processing is not strongest in the most face-selective voxels for each individual. Instead, the focus of the training effect occurred in voxels neighboring and overlapping to some extent with each participant's rFFA, without any systematic spatial relationship between the two. The individually defined letter- and object-selective regions did not show any significant training effect or correlation with configural processing.

### Ventral Occipito-Temporal Cortex

**Figure 4:**
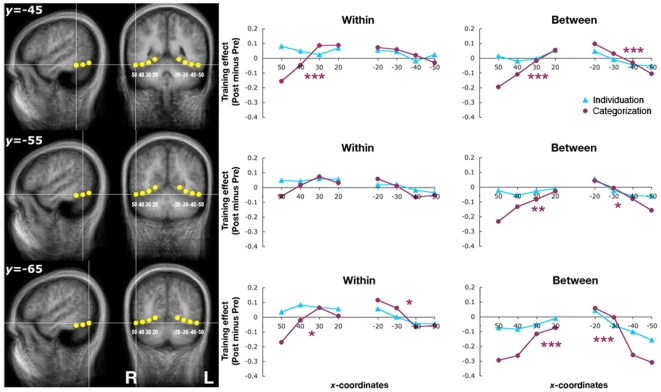
Training effects for the ventral occipito-temporal regions. The categorization group showed increased activity for the Ziggerins in the medial regions and decreased activity at the lateral regions after training, as indicated by the significance of the linear trends within each hemisphere (*p*<.05, .01, and .001 indicated by one, two, and three asterisks respectively). The individuation group did not show any reliable pattern of changes. The brain was depicted with a radiological convention (i.e., left hemisphere one the right).

Guided by the visual activations during the localizer scans, we localized a set of 24 ROIs positioned along VOT [15], [34]. There was a distributed pattern of training-induced changes associated only with categorization training, with increased activity in the medial regions and decreased activity towards the lateral regions (Fig. 4). For instance, for the between-class discrimination condition at y = −45, categorization training effect was positive at the medial regions (*y* = 20 on the right hemisphere and *y* = −20 on the left hemisphere) and gradually became negative towards the lateral regions (*y* = 40, 50 on the right hemisphere and *y* = −40, −50 on the left hemisphere). To examine this pattern, we conducted linear trend analyses for the ROIs as a function of their distance from the midline within each hemisphere at each *y*-coordinate (−45, −55, −65). For the categorization-training group, significant linear relationships were observed between the distance from the midline and the training effect in both hemispheres, especially for the between-class discrimination (within: *P*<.001 at *y* = −45, right hemisphere, *P*<.05 at *y* = −65, left hemisphere; between: *P*<.001 at *y* = −45 and −65, both hemispheres, *P*<.01 at *y* = −55, right hemisphere; *P*<.05 at *y* = −55, left hemisphere). Such linear trends did not occur systematically for the individuation-training group.

Consistent with the trend analyses, one-sample *t*-tests (*P*<.05) showed that, for the categorization-training group, there were positive training effects in some medial ROIs [(*x*,*y*) = (30,−45) and (20,−65) for within-class discrimination] and negative training effects in lateral ROIs [(*x*,*y*) = (40,−45), (50,−45), (40,−55), (50,−55), (−50,−65), (−40,−65), (30,−65), (40,−65), and (50,−65), all for between-class discrimination]. No significant training effect was found in any of the ROIs for the individuation-training group. A Group (individuation vs. categorization training)×*x*-coordinate (50, 40, 30, 20, −20, −30, −40, −50) ANOVA also showed a significant Group×*x*-coordinate interaction for ROIs at *y* = −45 [within: *F*_7,112_ = 2.13, *P* = .045; between: *F*_7,112_ = 2.22, *P* = .037]. Further analyses showed that the training effects were different across the medial and lateral regions only for the categorization-training group [within: *F*_7,56_ = 3.55, *P* = .003; between: *F*_7,56_ = 4.75, *P*<.001] but not for the individuation-training group [within: *F*_7,56_<1; between: *F*_7,56_ = 1.18, *P* = .323].

Notably, two ROIs [(*x*,*y*) = (40, −45), (50, −45)] close to the right fusiform region (40, −44, −18) identified in the localizer scans showed a trend of training effects for the individuation-training group that did not reach significance (*P*s>.40). However, there were significant correlations between the training effects in these two regions and the configural processing effect measured behaviorally [for (*x*,*y*) = (40, −45), within: *r* = .59, *P*<.01; between: *r* = .50, *P* = .03; for (*x*,*y*) = (50, −45), within: *r* = .78, *P*<.0001; between: *r* = .54, *P* = .01;], similar to that found for the right fusiform region. Such a correlation did not exist for any other ROI.

## Discussion

Our results show that different kinds of learning experience about objects causes different kinds of changes in activity within visual cortex. Learning to individuate Ziggerins increased activity for Ziggerins in a right fusiform region, with the increase correlated with configural processing of the Ziggerins measured outside the scanner [2]. In contrast, learning to categorize Ziggerins at the basic level resulted in a more distributed pattern of changes, with increased activity for Ziggerins at the medial parts of the VOT relative to lateral parts.

Interestingly, the type of testing tasks used in the scanner also revealed different levels of neural activity depending on the kind of training the participant received. Specifically, the effects of individuation training in the right fusiform were more conspicuous in within-class discrimination while effects of categorization training in the medial VOT were more conspicuous in between-class discrimination. This result suggests that patterns of activity in visual cortex following prolonged training with objects do not always emerge automatically but depend on the nature of the testing task. While this contrasts with previous novel object training studies [e.g., 1] that were able to show neural effects using a passive viewing task in the scanner, an important caveat is that earlier work involved significantly more training on a family of homogenous objects. So it remains open whether significantly more training with Ziggerins, resulting in higher levels of automaticity, would result in task-independent engagement of visual cortex by those objects. That said, one implication of our results is that future research examining qualitative differences in neural activity following different training regimens may want to include testing conditions that favor the transfer from each kind of training.

The stark contrast in both behavioral and neural measures following individuation versus categorization training offers additional support for the process-map hypothesis [35], [36]. According to this hypothesis, activity in visual cortex in response to objects reflects the demands of prior learning conditions. The VOT contains regions with different pre-existing biases, or preference for specific types of task demand. With repeated experience performing particular tasks with an object category during learning, regions best suited to those tasks may become automatically engaged by those objects in the future. Certainly, it is unlikely that one factor would suffice to account for the selective activity patterns for different object categories. Types of experience may work in concert with other factors, such as shape [18], [21] or connectivity with regions outside visual cortex [37] to govern which parts of VOT will be most engaged by a given category [35], [38], [39].

Despite the dissociated patterns of neural effects, the two training regimens did not result in training effects directly within the face-selective rFFA and the letter-selective region, despite the fact that they were modeled after face and letter processing respectively. This could be due to the insufficient amount of learning experience in this study, or to additional factors that are required in the formation of face-like and letter-like activity patterns. Individuation training effects have been obtained near but not directly within the FFA proper [see 5 for a similar finding]. This could indicate an intermediate level of individuation expertise. Because of the requirements of our design, participants learned to individuate objects from six different basic-level classes, compared to only one class in prior work on individuation training that recruited the FFA [1] that had the same training duration. Our participants thus received about 1/6^th^ of the experience with each category compared to prior work, and of course considerably less experience than any kind of real-world expertise shown to recruit the FFA [23], [40], [41].

Our categorization training, modeled after some aspects of the experience we have with letters, did not change activity in the letter-selective area. We should note that there is a high degree of variability of letter selectivity across individuals and across studies [10], which raises the bar on the ability to unambiguously detect letter-like selectivity with novel objects. Furthermore, our relatively short training regimen, and the smaller number of Ziggerin classes compared to the number of letters we learn in the real world, might also weaken the ability to find letter-like selectivity. One recent training study using pseudoletters showed letter-selective activity patterns only after training that involved writing but not typing or only visual experience [42], so combining two-dimensional letter-like stimuli plus sensorimotor learning may be key to recreate letter-like neural activity.

One interpretation of our current results is that the medial object regions were engaged more after categorization training because of their preference for peripheral, low-resolution representations, as compared to the lateral regions that have been tied more to foveal, high-resolution representations [39]. It remains to be seen whether the reduced activity at the lateral regions actually indicates less engagement or changed response tuning. Understanding the differential involvement of medial and lateral regions of visual cortex following training may ultimately require designs that permit multi-voxel pattern analyses [43], which go beyond analyzing average response within a region to probing distributed activity patterns across voxels.

In conclusion, despite using relatively short training regimens, participants in our studies showed qualitatively different perceptual strategies in their behaviors (Wong et al., 2009) and qualitatively different patterns of neural activity in visual cortex. Our work demonstrates that the demands of prior learning experience with a category of objects is one important factor governing the spatial distribution of neural changes when we acquire new visual object representations.

## Materials and Methods

### Participants

Eighteen volunteers participated in two fMRI sessions, one before and one after training. Nine were in the individuation-training group (six females, seven right-handed, age M = 22.11, SD = 1.32) and nine in the categorization-training group (five females, six right-handed, age M = 21.22, SD = 1.22). They received $12 for each behavioral session and $25 for each fMRI session. These subjects were randomly selected from those tested behaviorally by Wong et al. [30]. All had normal or corrected vision and reported no history of neurological disorders.

### Stimuli and Material

Seventy-two novel objects (Ziggerins in Fig. 1a) were created using Carrara 5 software (DAZ Productions, Inc., http://www.daz3d.com). There were six classes of Ziggerins, each defined by a unique part structure. Within each class, there were 12 styles, each defined by variations in the parts' cross-sectional shape, size, and aspect ratio. The same style variations applied across all six classes. This combination of class and style is analogous to six different letters in 12 different fonts. Each participant was trained on a subset of 36 Ziggerins (6 styles; selection randomized across participants), with the remaining Ziggerins reserved for pre- and post-training scans.

For fMRI scans, 72 Ziggerins (36 in trained set and 36 in transfer set) and 36 familiar object images (in six classes: beds, boats, cars, chairs, lamps, and teapots) were used. Images spanned a visual angle of 3.8° during training and 4° during scanning. In separate localizer scans, 144 grayscale images including 36 faces (half female), 36 familiar objects (those not used in the Ziggerin scans), 36 Roman letters (all except c, i, j, l, o, v, x, and z; in two fonts), and 36 pseudoletters (formed by rearranging the strokes of each Roman letter) were used.

All training and testing was conducted on Mac computers using MATLAB™ (MathWorks, Natick MA) with the Psychophysics Toolbox extension [44], [45].

### Training

Training occurred over ten one-hour sessions between the two fMRI scans. Participants in the *individuation-training* group learned individual names (two-syllable nonsense words) of Ziggerins in three tasks: naming, verification, and matching (Fig. 1b). In naming, participants entered the first letter of the name of the Ziggerin shown. In verification, participants verified if a name matched with a Ziggerin. In matching, participants judged which one of two Ziggerins matched a name.

Participants in the *categorization-training* group learned to recognize the Ziggerins by naming at the class level and by rapidly categorizing Ziggerins in an array of other Ziggerins of the same style. Learning proceeded through three tasks: naming, verification, and matrix scanning. The naming and verification tasks were similar to those for individuation training, but used class names instead of individual names. In matrix-scanning (Fig. 1c), participants performed a guided visual search in a matrix of 40 Ziggerins. The training procedures and behavioral training effects are detailed in Wong et al. [30].

### Pre- and Post-Training fMRI Scans

Each participant underwent an fMRI session before and after training. There were six Ziggerin scans in both pre- and post-training sessions and three localizer scans only in the post-training session. A block design was used with different types of stimuli presented in separate blocks. Participants performed a one-back task, pressing a button with their right index finger only when two identical images appeared consecutively. Only 1/12^th^ of the trials required a response. Each trial began with a blank for 275 ms followed by the stimulus for 725 ms. Presentation of stimuli was randomized within blocks, and the presentation of blocks was counterbalanced across scans and participants.

In each *localizer scan*, each of the four conditions (faces, objects, letters, pseudoletters) appeared in four blocks of 16 seconds. Eight-second fixations were inserted after each cycle of the four conditions. In each *Ziggerin scan*, each of the three conditions appeared in six blocks of 12 seconds (Fig. 2). In the within-class discrimination condition, each block contained only Ziggerins within a single class, thus requiring subordinate-level discrimination to perform the one-back task. In the between-class condition, each block contained Ziggerins across different classes, thus requiring basic-level discrimination. In the object control condition, each block contained familiar objects from different categories. Neighboring blocks were separated by 6 seconds of fixation. Three scans were devoted to trained Ziggerins and three scans were devoted to new transfer Ziggerins.

### Imaging Parameters and Analyses

Imaging was performed using a 3-T Philips Intera Achieva MRI scanner and a gradient-echo echo-planar imaging sequence sensitive to brain oxygen-level dependent (BOLD) contrast (34 contiguous axial slices, 3×3×3 mm voxel size; TR = 2 sec).

High-resolution T1-weighted anatomical volumes were acquired using a 3-D Turbo Field Echo (TFE) acquisition (170 contiguous axial slices, 1×1×1 mm voxel size, TR = 8.9 ms). Data analyses, performed with Brain Voyager™ (www.brainvoyager.com), included 3D motion correction, temporal filtering (3 cycles/scan high-pass), spatial smoothing (6-mm FWHM Gaussian), and multi-study GLM (general linear model). Within each participant, all functional images in the pre- and post-training scans were co-registered to the anatomical images obtained during the pre-training scans.

Data from the localizer scans were used to identify regions selective for faces, letters, and objects. At the group level, activations for different conditions were compared using random effects analyses with a threshold of *p*<.001 (uncorrected). At the individual level, the same conditions were compared using fixed effects analyses with a threshold of *p*(FDR)<.05. A set of 24 ROIs was defined along a large portion of VOT activated by all conditions (faces, letters, objects, pseudoletters) compared with fixation baseline. These were 10×10×15-mm ROIs situated at different points along the anterior-posterior axis (*y*-coordinate = −45, −55, or −65) and the medial-lateral axis (*x*-coordinate = −20, −30, −40, or −50 on the left and 20, 30, 40, or 50 on the right). The location and extent of these ROIs along the *z*-axis was adjusted for each participant to ensure the best coverage of ventral cortex. Activation in each ROI was calculated only for voxels falling on the gray matter.

For Ziggerin scans, training effects in functional and anatomical ROIs were calculated by averaging activity for the fourth volume onwards in each block for Ziggerins relative to the Object control during the post- vs. pre-training scan. No systematic differences were found between trained and transfer scans in different analyses so they were collapsed in the report of results.

## Supporting Information

Supporting Information S1 (0.33 MB DOC)
