# Supplementary material for: Beyond Shape: How You Learn about Objects Affects How They Are Represented in Visual Cortex
Source: PLoS One. 2009 Dec 22;4(12):e8405. doi: 10.1371/journal.pone.0008405 (PMC2794531; doi:10.1371/journal.pone.0008405)

**Supporting Information S1**

*Behavioral results of the Ziggerin runs*

The accuracy and response time data for the Ziggerin runs are shown in Table 1. In general, performances improved with training. This was confirmed by a Group × Pre/Post × Run Type (trained, transfer) × Condition (within, between, control) ANOVA, which showed a main effect of Pre/Post [RT: *F*_1,16_ = 9.57, *P* < .01; Proportion correct: *F*_1,16_ = 4.12, *P* = .0595]. No main effect of or interaction with Group was found.

Other effects included a significant Condition effect [RT: *F*_2,32_ = 20.13, *P <* .0001; Proportion correct: *F*_2,32_ = 21.96, *P* <.0001], but it interacted with Pre/Post [Proportion correct: *F*_2,32_ = 3.23, *P* = .0524] and Run Type [RT: *F*_2,32_ = 3.62, *P* < .05]. Scheffé tests (*P*<.05) showed that, while accuracy followed the order of object control > Between > Within before training, performance improved such that Between became as accurate as the object control condition. For the Condition × Run Type interaction, responses were faster for trained than transfer runs only for Within condition.

**Supplementary Table 1**

Behavioral performance for the Ziggerin runs.

|  | Individuation Training | | | Categorization Training | | |
| --- | --- | --- | --- | --- | --- | --- |
|  | % correct | RT (ms) | % false positive | % correct | RT (ms) | % false positive |
| *Pre-training* |  |  |  |  |  |  |
| Trained |  |  |  |  |  |  |
| Within | 82.3 | 563 | 1.59 | 79.4 | 570 | 2.82 |
| Between | 86.0 | 560 | 0.88 | 87.2 | 570 | 0.64 |
| Control | 91.8 | 540 | 0.82 | 89.7 | 551 | 0.82 |
| Transfer |  |  |  |  |  |  |
| Within | 82.8 | 564 | 1.47 | 79.1 | 580 | 2.02 |
| Between | 86.7 | 555 | 0.64 | 82.8 | 561 | 1.06 |
| Control | 93.5 | 538 | 0.29 | 93.1 | 546 | 0.60 |
|  |  |  |  |  |  |  |
| *Post-training* |  |  |  |  |  |  |
| Trained |  |  |  |  |  |  |
| Within | 91.7 | 542 | 1.29 | 77.9 | 544 | 2.94 |
| Between | 93.8 | 534 | 0.82 | 85.8 | 530 | 1.76 |
| Control | 90.5 | 522 | 0.47 | 90.7 | 518 | 0.49 |
| Transfer |  |  |  |  |  |  |
| Within | 88.4 | 559 | 1.06 | 81.6 | 556 | 2.32 |
| Between | 93.4 | 537 | 0.17 | 89.4 | 525 | 1.07 |
| Control | 93.0 | 518 | 0.52 | 90.0 | 527 | 0.58 |

**Supplementary Figure 1**

The face-selective area defined for all the participants as a group (enclosed by the white line) and the rFFAs defined for each individual in the individuation group (in different colors). The individually defined rFFA clustered around the group-defined area, though there was no perfect overlap (on average 20% of the group-defined area overlapped with the individually defined rFFA). The average Talairach coordinates of individual rFFAs (38.9, -43.4, -14.6) were very similar to those of the group-defined face-selective area (40, -44, -18), with an average distance of 7.6mm.


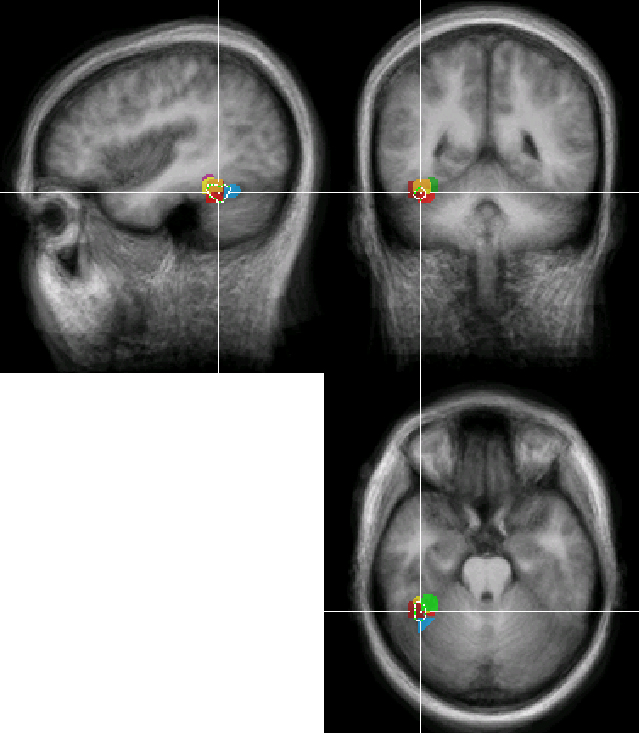

Supplement: Supporting Information S1 — (0.33 MB DOC) [file pone.0008405.s001.doc]
